# Longitudinal analysis of DC subsets in patients with ovarian cancer: Implications for immunotherapy

**DOI:** 10.3389/fimmu.2023.1119371

**Published:** 2023-02-10

**Authors:** Beatris Mastelic-Gavillet, Apostolos Sarivalasis, Leyder Elena Lozano, Sebastien Lofek, Tania Wyss, Ignacio Melero, I. Jolanda M. de Vries, Alexandre Harari, Pedro Romero, Lana Elias Kandalaft, Selena Viganó

**Affiliations:** ^1^ Department of Oncology, Centre Hospitalier Universitaire Vaudois and Lausanne University Hospital, Lausanne, Switzerland; ^2^ Ludwig Institute for Cancer Research, University of Lausanne, Lausanne, Switzerland; ^3^ Bioinformatics Core Facility, Swiss Institute of Bioinformatics (SIB), Lausanne, Switzerland; ^4^ Division of Immunology and Immunotherapy, Center for Applied Medical Research, University of Navarra, Pamplona, Spain; ^5^ Instituto de Investigacion Sanitaria de Navarra, Pamplona, Spain; ^6^ Departments of Immunology-Immunotherapy and Oncology, University Clinic, University of Navarra, Pamplona, Spain; ^7^ Program of Immunology and Immunotherapy, Centro de Investigacion Biomedica en Red Cancer, Madrid, Spain; ^8^ Department of Tumour Immunology, Radboud Institute of Molecular Life Sciences, Nijmegen, Netherlands

**Keywords:** DC, ovarian cancer, cancer vaccine, TLR3, chemotherapy

## Abstract

**Background:**

The use of circulating cDC1 to generate anti-cancer vaccines is among the most promising approaches to overcome the limited immunogenicity and clinical efficacy of monocyte-derived DC. However, the recurrent lymphopenia and the reduction of DC numbers and functionality in patients with cancer may represent an important limitation of such approach. In patients with ovarian cancer (OvC) that had received chemotherapy, we previously showed that cDC1 frequency and function were reduced.

**Methods:**

We recruited healthy donors (HD, n=7) and patients with OvC at diagnosis and undergoing interval debulking surgery (IDS, n=6), primary debulking surgery (PDS, n=6) or at relapse (n=8). We characterized longitudinally phenotypic and functional properties of peripheral DC subsets by multiparametric flow cytometry.

**Results:**

We show that the frequency of cDC1 and the total CD141+ DC capacity to take up antigen are not reduced at the diagnosis, while their TLR3 responsiveness is partially impaired in comparison with HD. Chemotherapy causes cDC1 depletion and increase in cDC2 frequency, but mainly in patients belonging to the PDS group, while in the IDS group both total lymphocytes and cDC1 are preserved. The capacity of total CD141^+^ DC and cDC2 to take up antigen is not impacted by chemotherapy, while the activation capacity upon Poly(I:C) (TLR3L) stimulation is further decreased.

**Conclusions:**

Our study provides new information about the impact of chemotherapy on the immune system of patients with OvC and sheds a new light on the importance of considering timing with respect to chemotherapy when designing new vaccination strategies that aim at withdrawing or targeting specific DC subsets.

## Introduction

DC vaccination is a very promising approach for inducing specific immune responses against cancer. Indeed, despite the limited clinical efficacy observed so far, vaccines have the potential to improve cancer immunotherapy efficacy in combinatorial approaches by inducing specific recognition of tumor cells with little or no evidence of treatment-limiting toxicity (1). Recently, novel approaches aim at using circulating DC as an alternative to monocyte-derived DC that, so far, have not shown objective clinical antitumor activity. Two conventional DC (cDC) subsets that are important in developing anti-tumor T cell responses are found in the peripheral blood: cDC1s (CD141^bright^), which are fundamental for CD8 T cell activation, and cDC2s (CD1c^+^) which are key for CD4 T cell activation (2, 3). Among the conventional DC (cDC) subsets, cDC1 are the most promising for promoting antigen-specific tumor cell killing. Indeed, they are endowed with the highest cross-presentation capacity and secrete chemoattractant molecules for CD8 T cells (3–5). Of importance, cDC1 tumor infiltration has been associated with better clinical outcome in some tumor types (6, 7). Moreover, BATF3-/-_mice deficient in cDC1 do not respond to various types of immunotherapies (6, 8).

Ovarian cancer (OvC) is the most lethal gynecological cancer, with an overall 5-year-survival rate below 50%. Despite optimal first-line treatments including surgery and platinum-based chemotherapy, 75% will eventually relapse. These disappointing results are partially improved by recent advances in maintenance treatment including bevacizumab and poly-adenosine diphosphate-ribose polymerase (PARP) inhibitors. Despite these, only a subset of patients experiences long lasting clinical benefits. Interestingly, patients presenting high immune cell infiltrated tumors have an increased overall survival, thus suggesting potential for immunotherapy to mediate clinical benefit (9). However, the efficacy of current immune checkpoint inhibitors, such as the Pembrolizumab, a PD-1 inhibitor, showed an overall response rate of only 11.5% (10). Moreover, adoptive T cell therapy is hindered by frequent poor T cell infiltration and low antigen presenting function of infiltrating DC (11). Thus, new immunotherapeutic strategies need to increase the overall antigen presentation at the tumor site and improve T cell infiltration, throwing the light on DC-based vaccines.

The low frequency and severity of adverse events together with encouraging results from clinical trials, makes the use of DC vaccines a promising approach to improve clinical outcome in patients with OvC (12, 13). A main limitation, likely impacting the success of DC-based vaccines in cancer patients, is the alteration of lymphocyte number and function (6–8, 14, 15). Indeed, multiple clinical trials that combined DC vaccines with other immunotherapies and were targeting multiple antigens (pool of peptides or whole tumor lysates), showed clinical efficacy, but only in a subgroup of patients (13, 16–18). The use of circulating DC for vaccine strategies must also keep into consideration potential alterations in DC frequency and function. In patients with OvC, the frequency of DC subsets in ascites is not correlated with their survival (19). However, tumor-infiltrating DC are predominantly plasmacytoid and show a partially mature phenotype and display regulatory functions (20–24).

Of importance, we recently showed that the frequency and the function of cDC1 and total CD141^+^ DC in periphery is reduced in patients with OvC who already underwent treatment (25), rendering the use of such cells for vaccines difficult. However, information regarding the contribution of the disease, the stage and the treatment on the quantitative and qualitative characteristics of DC in patients with OvC is still missing. Addressing these points is pivotal to understand whether DC-based therapies for OvC would require optimized timing for DC isolation, or particular combination with strategies preventing/restoring DC number or function (6, 26, 27) or to use/target DC subsets triggering the most effective anti-tumor response.

In the present study, we characterized the phenotype and the function of immune subsets in the peripheral blood from patients with OvC at diagnosis (treated by interval debulking surgery (IDS), or primary debulking surgery (PDS)) or at recurrence (relapse group), and during treatment (Carboplatin with or without Paclitaxel + surgery). At diagnosis, cDC1 were not significantly lower in patients with OvC than in healthy donors (HD). However, both lymphocyte counts and cDC1 frequency were significantly reduced by chemotherapy in PDS and recurrence groups but not in the IDS group, which also presented a reduction in the PDL1 expression by CD141^+^ DC and stable expression of CD80. In addition, we show that the total CD141^+^ DC were not impaired in their capacity to capture the antigen in patients with OvC; however, for the first time, we indicated that among the CD141^+^ DC the most able to take the antigen are cells expressing CD86 and co-expressing ILT3 and ILT4. Finally, CD141^+^ DC in patients with OvC responded only partially to Poly(I:C) stimulation at diagnosis and this function was further reduced by chemotherapy.

Our study indicates that the IDS group had better preserved lymphocytes and cDC1 than the PDS group and sheds the light on new clinical implications when considering immunotherapy. In addition, the use of circulating DC drawn from OvC patients as vaccine may require to collect cDC1 before starting chemotherapy, to combine strategies aiming at improving response to TLR agonists and, eventually, to select subsets that have the highest antigen uptake potential.

## Materials and methods

### Subjects and specimen preparation

Human blood samples from HD and patients were collected in accordance with the declaration of Helsinki principles. Written informed consent was obtained from all HD and patients (protocols: 2016-02094 and 2019-00546). Blood draws in patients with OvC were taken at diagnosis/relapse, at 2 time points during treatment and at the end of treatment (last day of treatment). Clinical data and time points of blood draws from patients with OvC are described in **Table 1**.

**Table 1:** Clinical characteristics of the patients with OvC.

Pt	TP	Date (day,month, year)	Group	Age	FIGO Stage	BRCA	n° of lines	Best reponse to latest line	Delta last relapse (months)	Date of death	CA125 (kU/l)	Leucocyte count (G/l)
00IP	PreT	22.01.2020	IDS	75	IIIC	WT	1	NED	na	2021	283	8.8
00IP	PreD	30.03.2020									54	4.7
00IP	PostD	03.04.2020									96	4.9
00IP	EndT	17.06.2020									58	7.1
1G5G	PreT	15.01.2020	IDS	70	IVB	WT	1	PR	na	na	2336	7.2
1G5G	PreD	23.03.2020									119	3.8
1G5G	PostD	09.04.2020									na	8.2
1G5G	EndT	27.05.2020									na	5.1
1FRU	PreT	09.08.2019	IDS	75	IIIC	WT	1	PR	na	na	4886	9.2
1FRU	PreD	04.12.2019									73	1.9
1FRU	EndT	28.02.2020									17	4.9
1FQI	PreT	26.07.2019	IDS	75	IVA	WT	1	PR	na	na	3295	8.7
1FQI	PreD	25.09.2019									311	19.4
1FQI	PostD	22.11.2019									88	2.1
1FQI	EndT	23.01.2020									57	4.7
1CU4	PreT	14.09.2019	IDS	73	IIIC	WT	1	CR	na	na	109	4.6
1CU4	PreD	11.12.2019									51	4.4
1CU4	PostD	09.01.2020									19	3.3
1CU4	EndT	02.04.2020									15	3.5
1FIB	PreT	17.04.2019	IDS	79	IIIC	WT	1	CR	na	na	1382	8.8
1FIB	PreD	17.07.2019									177	8.5
1FIB	PostD	22.07.2019									177	5.7
1FIB	EndT	25.09.2019									164	4.2
1EW1	PreD	06.03.2019	PDS	67	IIIA1	WT	1	PR	na	na	179	4.2
1EW1	PreT	05.04.2019									70	3.8
1EW1	MidT	07.06.2019									34	1.6
1EW1	EndT	02.08.2019									39	2.7
18GV	PreD	04.07.2018	PDS	66	IIIA2	WT	1	PR	na	na	1097	8.7
18GV	MidT	17.07.2018									1097	5.9
18GV	EndT	21.11.2018									43	2.2
1FJA	PreD	17.05.2019	PDS	68	IVB	WT	1	PR	na	na	365	6.8
1FJA	MidT	28.06.2019									27	4
1FJA	EndT	30.08.2019									20	3
1AK9	PreD	12.10.2018	PDS	58	IIIB	WT	1	PR	na	na	32	8.6
1AK9	MidT	19.12.2018									5	2.8
1AK9	EndT	23.01.2019									5	3.6
1FQJ	PreD	24.07.2019	PDS	38	IIIA2	WT	1	CR	na	na	136	5.4
1FQJ	MidT	04.09.2019									14	2.3
1FQJ	EndT	13.11.2019									10	3.6
1FS9	PreD	14.08.2019	PDS	60	IVB	WT	1	PR	na	na	93	9.5
1FS9	PreT	11.09.2019									111	8.8
1FS9	EndT	15.01.2020									8	4.6
OXLZ	PreR	16.08.2017	Relapse	50	IIIC	WT	2	PR	15	na	8	3.8
OXLZ	PreT	23.01.2019									205	7.8
OXLZ	MidT	06.03.2019									13	5
OXLZ	EndT	03.04.2019									10	19.2
OS5K	PreR	30.08.2017	Relapse	77	IVB	WT	2	PR	28	2020	25	5.4
OS5K	PreT	14.12.2018									1798	5.9
OS5K	MidT	06.02.2019									78	2.9
OS5K	EndT	01.05.2019									73	15.7
0TRA	PreT	13.03.2019	Relapse	80	IVB	WT	3	PD	3	2019	79	7.4
0TRA	MidT	03.04.2019									79	8.5
0TRA	EndT	08.05.2019									122	11.7
261	PreT	13.03.2019	Relapse	67	IIIB	WT	3	PR	2	na	22	5.8
261	MidT	12.04.2019									20	2.4
261	EndT	16.08.2019									16	2.7
14W7	PreT	08.03.2019	Relapse	51	IIIA2	WT	3	PD	4	2020	134	3.3
14W7	MidT	17.04.2019									113	3.6
14W7	EndT	25.06.2019									223	3.6
OYDN	PreR	30.08.2017	Relapse	49	IIIC	MUT	5	PR	19	na	6	2.3
OYDN	PreT	24.04.2019									931	4.2
OYDN	MidT	14.08.2019									94	1.1
OYDN	EndT	11.09.2019									68	3.1
14Z5	PreR	21.03.2018	Relapse	65	IVB	WT	2	PR	8	2020	2827	7.3
14Z5	PreT	03.05.2019									244	na
14Z5	MidT	09.07.2019									170	3.5
14Z5	EndT	26.09.2019									30	3.4
1CD5	PreT	11.12.2019	Relapse	62	IVA	WT	2	PR	2	2021	110	6.5
1CD5	MidT	11.02.2020									48	22.9
1CD5	EndT	14.05.2020									13	6.3
0X7Z	PreR	20.12.2016	Relapse	62	IIIC	WT	4	PR	0	2021	na	5.8
0X7Z	PreT	08.01.2020									38	5
0X7Z	MidT	18.03.2020									33	5.4
0X7Z	EndT	20.05.2020									14	4.8
1E7E	PreT	22.01.2020	Relapse	84	IVB	WT	3	PD	2	2020	134	7.2
1E7E	MidT	18.03.2020									1017	4.3
1E7E	EndT	20.05.2020									722	5.9

### PBMC isolation

Fresh anticoagulated blood diluted at a 1:2 ratio in PBS was layered on lymphoprep (ratio of diluted blood:lymphoprep 1.5:1). Mononuclear cells were isolated by density gradient centrifugation (1800 rpm, 20 min centrifugation without break, room temperature), washed twice and immediately cryopreserved in 90% fetal calf serum (FCS) and 10% DMSO.

### Culture media

RPMI 1640 Glutamax Supplement (Thermo Fisher Scientific, Waltham, MA, United States), 10% Human Serum (BIOWEST, Riverside, MO, United States), 1mM Na pyruvate (Thermo Fisher Scientific, Waltham, MA, United States), 10mM/ml Hepes (Thermo Fisher Scientific, Waltham, MA, United States), 1X MEM Non-Essential Amino Acids (Thermo Fisher Scientific, Waltham, MA, United States), 0.1% β-mercaptoethanol and 1% penicillin-streptomycin (Bioconcept, Allschwil/BL, Switzerland).

### Antibodies and reagents

Anti-CD11c BV650 (clone 3.9), anti-CD80 PeCy5 (clone 2D10), anti-CD1c BV510 (clone L161), anti-HLA-DR BV570 (clone L243), anti-XCR1 PE (clone S15046E), anti-CD40 BV605 (clone 5C3), anti-CD303 (BDCA2) PE/Dazzle 594 (201A), anti-CD3 BV421 (clone UCHT1), anti-CD19 BV421 (clone HIB19), anti-ILT4 PE (clone 42D1), anti-ILT3 PE-Cy7 (clone ZM4.1), were purchased from BioLegend (San Diego, CA, United States). Anti-Clec9a VioBright-FITC (clone 8F9), anti-CD123 APC-Vio770 (clone AC145), anti-CD141 APC were purchased from Miltenyi Biotec (Bergisch Gladbach, Germany). Anti-CD274 (PDL1) PE-Cy7 (clone MIH1), anti-CD86 AlexaFluor 700 (clone 2331), Anti-CD276 BV711 (clone 232-5), anti-CD14 PB (clone M5E2) were purchased from BD-Biosciences (Franklin Lakes, NJ United States). RBC lysis solution was purchased from Qiagen (Hilden, Germany). FcR blocking reagent was purchased from Miltenyi (Bergisch Gladbach, Germany). Poly(I:C) HMW was purchased from *In vivo*gen (San Diego, CA, United States). Fluorescein isothiocyanate-dextran was bought from Sigma-Aldrich.

### Flow cytometry analyses

#### Fluorescein isothiocyanate-dextran staining

When indicated cells were incubated for 30 minutes at 4°C or 37°C with 1mg/ml of fluorescein isothiocyanate-dextran in 24 wells plates (2-4 x10^6^ cells/well), then washed with cold PBS and before proceeding with surface staining.

#### Poly(I:C) stimulation

When indicated cells were incubated for 16-18 hours in 24 wells plates (1-2 x10^6^ cells/well) at 37°C with or without 20 µg/ml of Poly (I:C). Cells were collected, stained, and analyzed by flow cytometry.

#### Flow cytometry staining and data analysis

Cells were incubated for 10min with FcR blocking reagent and stained in PBS-EDTA with the appropriate antibodies.

Flow cytometry acquisition was performed with an LSR Fortessa flow cytometer (BD Biosciences, Franklin Lakes, NJ United States). Flow cytometry analysis was performed with FlowJo software (Version 10.2, Treestar). Data were analyzed by Prism v7.

### Statistical analysis

Statistical analysis was performed with Prism software (Version 7, GraphPad). Statistically significant differences among multiple groups and adjusted *p*-values were calculated by one-way ANOVA followed by pairwise comparisons using Dunn’s test. Rank correlations were performed by using the nonparametric Spearman’s test to determine the Spearman’s rank correlation coefficients (r) and their associated *p*-values. A *p*-value of 0.05 or lower was considered as indicating a statistically significant difference.

## Results

### Patients with OvC in the IDS cohort have lower chemotherapy-induced lymphopenia and less loss of cDC1

Recently, we showed that the frequency of cDC1 is reduced in patients with OvC (25). However, the impact of cancer-related pathogenesis *vs* therapeutic intervention (chemotherapy and surgery) on the frequency of DC subsets was not dissected. Thus, we recruited three groups of patients with different clinical characteristics (**Table 1** and **Supplementary Figure 1A**). Patients recruited at diagnosis who could not have surgery received neoadjuvant chemotherapy before surgery (IDS group), while the second group of patients was treated with chemotherapy after undergoing debulking surgery (PDS group); the third group was composed of patients already treated in the past but in which the cancer recurred (relapse). The recruitment was done at diagnosis (IDS and PDS) or at time of relapse and samples were collected at four time points (**Table 1** and **Supplementary Figure 1A**).

**Figure 1:**
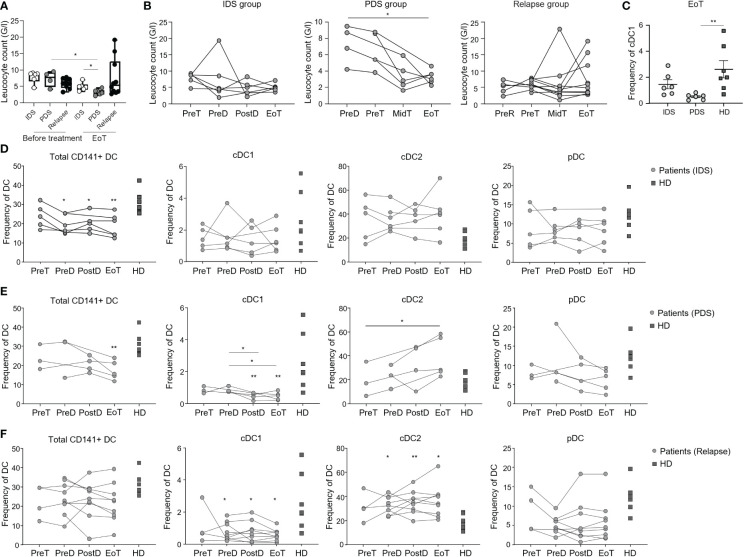
Longitudinal monitoring of lymphocytes and DC populations in patients with OvC and HD. **(A)** Lymphocyte counts in patients with OvC measured at recruitment or at the end of treatment (EoT). White dots represent patients undergoing IDS regimen, grey dots represent patients undergoing PDS regimen, while black dots represent relapsing patients. The box encompasses the interquartile range, the central band indicates the median, and the whiskers reach the minimum and maximum values. **(B)** Lymphocyte counts in patients with OvC measured at recruitment, during treatment and at EoT. **(C)** Frequency of cDC1 in patients with OvC belonging to IDS or PDS groups at the end of treatment and in HD. Cumulative data of the frequency of DC subsets in HD (dark grey squares) and in patients with OvC (grey dots) belonging to **(D)** IDS group, **(E)** PDS group and **(F)** relapse group. *p < 0.05, **p < 0.01. One-way ANOVA tests followed by pairwise Dunn’s tests.

To date, there is no clear indication of whether IDS is to be preferred to PDS as standard of care. However, IDS has been reported to reduce the postoperative rates of adverse effects and mortality (28, 29) and is recommended in patients with high tumor burden or worse clinical presentation. Consistent with the literature (30), in our study, patients belonging to the IDS group in our study showed the highest circulating CA125 levels (**Table 1** and **Supplementary Figure 1B**). In all groups, however, CA125 decreased upon treatment (**Supplementary Figure 1C**). On the other hand, lymphocyte counts did not differ between IDS and PDS groups, while they tended to be lower in relapsing patients, likely due to previous treatments (**Figure 1A**). Of interest, upon initiation of chemotherapy the lymphocyte count was more strongly reduced in the PDS group than in the IDS group (**Figures 1A, B**) leading to a significant difference in lymphopenia at the end of therapy (**Figure 1A**). These data may have important implications for immunotherapy efficacy as they suggest a better immunological state in patients belonging to the IDS group compared to the PDS group.

In the present study, we comprehensively analyzed by flow cytometry the frequency of different lymphocytic- and monocytic-cell subsets in the peripheral blood of the enrolled patients at different time points and in HD (age- and sex-matched). We found that chemotherapy led to a statistically significant reduction of T cell frequency only in the relapsing group (**Supplementary Figure 1D**). In addition, the ratio CD4 to CD8 T cells was higher in the IDS group before treatment than in HD or other patient groups, but this was partially reduced upon treatment (**Supplementary Figure 1E**). Finally, we found an increased proportion of circulating classical monocytes in patients with OvC when compared to HD (**Supplementary Figures 1F, I**).

Next, we monitored the frequency of DC populations (*i.e.*, cDC1, cDC2, pDC) as well as of the total CD141^+^ DC (**Supplementary Figure 1J**) in patients with OvC and in HD. Consistently with our previous findings (25), we observed a reduction in the frequency of cDC1 and an increase in cDC2 in relapsing patients in comparison with HD (**Figure 1F**). Of importance, this difference was not present at diagnosis for the IDS and PDS groups (**Figures 1D, E**). However, upon initiation of chemotherapy, only patients in the PDS group had a statistically significant loss of cDC1 (**Figures 1D, E**) that became lower in frequency than the ones measured in HD (**Figure 1C**). Along the same line, only patients in the PDS group had an increase in cDC2 (**Figures 1D, E**) even though we did not find a statistical difference with the HD (**Figure 1E**). These data indicate a leading role for chemotherapy in depleting cross-presenting DC, but also suggest that lymphocyte loss is prevented in the IDS group.

Due to their relevance in antigen uptake and presentation capacity, and homing (31–33), we evaluated the expression of CLEC9A and XCR1 by the cDC1. No difference was observed between patients with OvC and HD or among the different time points of blood sampling. (**Supplementary Figures 1K–M**).

**Figure 2:**
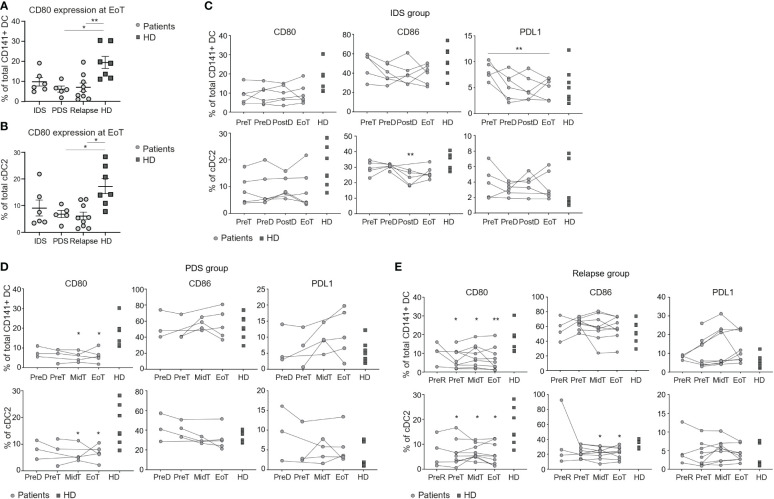
Longitudinal monitoring of the activation profile of DC populations in patients with OvC and HD. Cumulative data of the CD80 expression levels in total CD141^+^ DCs **(A)** and in cDC2 **(B)** measured in patients with OvC at the end of treatment and in HD. Cumulative data of the CD80, CD86 and PDL1 expression levels in total CD141^+^ DCs and in cDC2. Measurements were performed in patients with OvC (grey dots) at recruitment, during treatment and at EoT, belonging to **(C)** IDS group, **(D)** PDS group, or **(E)** relapse group and are compared to HD (dark grey squares). In the relapse group, analyses have also been performed at a time-point before time of relapse (PreR). *p < 0.05, **p < 0.01. One-way ANOVA tests followed by pairwise Dunn’s tests.

We then evaluated the costimulatory molecule expression profile by the different DC subsets in patients having OvC (**Supplementary Figure 2A**). Because the frequency of cDC1 and pDC subsets was too low to perform this in-depth analysis, we only considered total CD141^+^ DC and cDC2. CD80 expression by total CD141^+^ DC and cDC2 was significantly reduced in the PDS group and in relapsing patients (**Figures 2A, B, D, E**), but not in the IDS group (**Figures 2A–C**). On the other hand, PDL1 expression by total CD141^+^ DC was reduced during treatment in the IDS group (**Figure 2A**), reaching significant lower levels at EoT when compared to the relapse group (**Supplementary Figure 2B**).

### Total CD141^+^ DC expressing CD86 or ILT3^+^/ILT4^+^ are the most proficient in antigen capture

**Figure 3:**
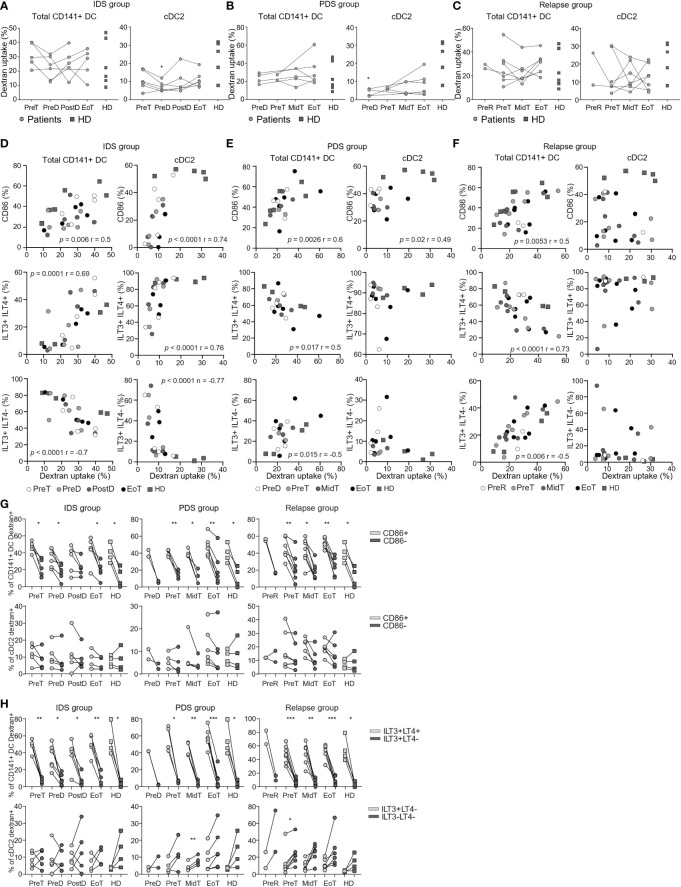
Longitudinal monitoring of the capacity to uptake dextran *in vitro* by DC populations in patients with OvC and in HD. Cumulative data of the frequency of dextran positive total CD141^+^ DC and cDC2. Measurements were performed in patients with OvC (grey dots) at recruitment, during treatment and at EoT, and compared to HD (dark grey squares). We show data from patients undergoing **(A)** IDS treatment regimen, **(B)** PDS treatment regimen and **(C)** relapsing patients. *p < 0.05, ** *p* < 0.01. One-way ANOVA tests followed by pairwise Dunn’s tests. Correlations between the frequency of dextran positive total CD141^+^ DC and cDC2 and the frequency of total CD141^+^ DC positive for CD86, ILT3 and/or ILT4 or the frequency of cDC2 positive for CD86, ILT3 only or negative for both ILT3 and ILT4. Correlations were performed with data derived from HD and patients undergoing **(D)** IDS treatment regimen, **(E)** PDS treatment regimen and **(F)** relapse. Spearman’s rank correlation tests were performed. **(G)** Cumulative data of the frequency of dextran positive total CD141^+^ DC/CD86^+^ (light grey symbols) or CD141^+^ DC/CD86^-^ (dark grey symbols) and cDC2/CD86^+^ (light grey symbols) or cDC2/CD86^-^ (dark grey symbols) measured in HD and in patients with OvC belonging to the IDS, PDS or relapse group. **(H)** Cumulative data of the frequency of dextran positive total CD141^+^ DC/ILT3^+^ILT4^+^ (light grey symbols) or CD141^+^ DC/ILT3^+^ILT4^-^ (dark grey symbols) and cDC2/ILT3^+^ILT4^-^ (light grey symbols) or cDC2/ILT3^-^ILT4^-^ (dark grey symbols) measured in HD and in patients with OvC belonging to the IDS or PDS or relapse group. *p < 0.05, **p < 0.01. One-way ANOVA tests followed by pairwise Dunn’s tests. ***p < 0.001.

We measured the capacity of total CD141^+^ DC and cDC2 to take up antigens by FITC-dextran uptake assay. This assay encompasses the quantification of both the micropinocytosis and the mannose receptor-mediated endocytosis capacity by DC and monocytes and it has been previously used to measure extracellular antigen capture (34–36). For these next assays, we only considered total CD141^+^ DC and cDC2 since the frequency of cDC1 and pDC subsets was too low to dissect them. Consistently with previous studies (37), total CD141^+^ DC tended to be better than cDC2 in taking up antigen, however this difference did not reach statistical significance probably due to low number of samples analyzed (data not shown). We did not observe differences in the overall capacity to take up antigens between patients and HD, or between different time-points in blood of patients upon initiation of treatment (**Figures 3A–C**).

DC are characterized by the expression of costimulatory (e.g., CD86) and inhibitory molecules (e.g., ILT3, ILT4) that can be regulated by DAMP and PAMP signaling. These molecules are also expressed at resting conditions; thus, we investigated their impact on DC function *ex vivo*. We measured the expression of ILT3 and ILT4 on total CD141^+^ DC and cDC2 (**Supplementary Figure 3A**). Firstly, we showed that total CD141^+^ DC are mostly ILT3^+^ILT4^+^ and ILT3^+^ILT4^-^, while cDC2 were mostly only ILT3^+^ or negative for these molecules (**Supplementary Figures 3A, B**). In addition, the activation status of DC was not different between patients with OvC and HD, and did not change in patients during treatment (**Supplementary Figures 3C–E**). Then, we investigated whether there was a specific DC activation status principally responsible for antigen uptake (**Supplementary Figures 3F, G**). Interestingly, we found a positive association between the expression of CD86 and the capacity of both DC subsets to take up antigens in the IDS and PDS groups (**Figures 3D, E**). This association was also found for the total CD141^+^ DC in the relapse group (**Figure 3F**). In addition, the expression of ILT3 and ILT4 was also influencing the DC capacity to capture antigens. We found that the antigen uptake capacity of total CD141^+^ DC correlated directly with the frequency of ILT3^+^ILT4^+^ CD141^+^ DC, while it correlated indirectly with the frequency of total CD141^+^ DC expressing only ILT3 (**Figures 3D–F**). However, for cDC2 and only in the IDS cohort, the antigen uptake capacity was directly correlated with the frequency ILT3^+^ILT4^-^ cDC2, while it correlated indirectly with the frequency of cDC2 expressing none of these molecules (**Figures 3D–F**). Importantly and consistently with these correlations, the CD86^+^ CD141^+^ DC were more able to take up antigen in comparison to the CD86^-^ CD141^+^ DC (**Figure 3G**). In addition, ILT3^+^ILT4^+^ CD141^+^ DC were more able to take up antigen than the total CD141^+^ DC expressing only ILT3 (**Figure 3H**). This was not observed when comparing the ability of CD86^+^ and CD86^-^ cDC2, or ILT3^+^ILT4^-^ and ILT3^-^ILT4^-^ cDC2 to take up the dextran (**Figures 3G, H**).

### Total CD141^+^ DC response to Poly(I:C) is impaired in patients with OvC and it is worsened by chemotherapy

**Figure 4:**
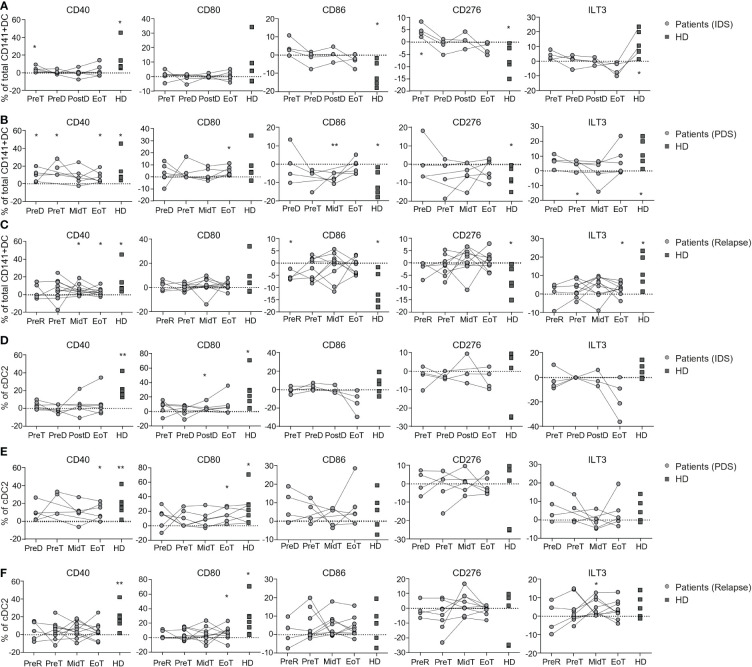
Longitudinal monitoring of the capacity to respond to Poly(I:C) *in vitro* by DC populations in patients with OvC and in HD. Cumulative data of the variation (frequency subtraction from the unstimulated cells) in the expression of CD40, CD80, CD86, CD276 and ILT3 by total CD141^+^ DC after Poly(I:C) stimulation in HD (dark grey squares) and in patients (grey dots) belonging to **(A)** IDS, **(B)** PDS, or **(C)** relapse group. Cumulative data of the variation (frequency subtraction from the unstimulated cells) in the expression of CD40, CD80 CD86, CD276 and ILT3 by cDC2 after Poly(I:C) stimulation in HD (dark grey squares) and in patients (grey dots) belonging to **(D)** IDS, **(E)** PDS or, **(F)** relapse group. *p < 0.05, **p < 0.01. One-way ANOVA tests followed by pairwise Dunn’s tests. Statistical differences refer to comparison with the unstimulated control.

To prime a proper adaptive immune response against a specific antigen, DC need to mature through TLR and/or inflammasome signaling mediated by PAMPs and DAMPs. Mature DC express costimulatory molecules required for T cell priming (e.g., CD40, CD80 and CD86) as well as inhibitory molecules required to avoid excessive immune responses (e.g., PDL1, CD276, and ILT3). We previously observed that total CD141^+^ DCs in patients with OvC were less responsive to TLR3 stimulation (Poly(I:C)) than total CD141^+^ DCs in HD (25). However, the impact of chemotherapy on the capacity of DC to respond to Poly(I:C) has not been addressed. Thus, we investigated the capacity of DC subsets from OvC patients and HD to respond to Poly(I:C) by measuring the change in expression of CD40, CD80, CD86, PDL1, CD276, and ILT3 (**Supplementary Figure 4**). Total CD141^+^ DC triggered directly *ex vivo*, responded to Poly(I:C) by increasing CD40 and ILT3, while decreasing CD86 and CD276 expression in HD (**Supplementary Figure 4** and **Figures 4A–C**). In patients with OvC, total CD141^+^ DC isolated before starting treatment increased the expression of CD40 in response to Poly(I:C) (both IDS and PDS groups), but they did not increase ILT3, CD86 or CD276 expression (**Figures 4A, B**). Upon initiation of treatment, however, the capacity to upregulate CD40 was also lost (**Figures 4A–C**). These data show that total CD141^+^ DC tend to be functionally impaired in patients with OvC already at diagnosis and this is worsened by the initiation of chemotherapy. The cDC2 subset responded to Poly(I:C) by increasing the expression of CD40 and CD80 in HD, but this response was weaker in all patient groups (**Figures 4D–F**), suggesting that the cDC2 subset may also be functionally impaired in patients with OvC independently of treatment.

## Discussion

Exploiting circulating cDC1 to generate cancer vaccines instead of monocyte-derived DC may give better immunogenicity and efficacy due to their better cross-presenting capacity and pro-inflammatory phenotype. However, in patients affected by OvC that were previously treated, this may be a challenge due to the reduced frequency and function of this DC subset (25). We therefore performed a detailed immunomonitoring and functional characterization of DC subsets in patients with OvC before, during, and after receiving chemotherapy to identify the optimal timing to harvest these cells.

Newly diagnosed treatment-naïve patients had comparable levels of cDC1 than HD, while, consistent with our previous findings (25), patients with OvC that were undergoing relapse had a reduced frequency of cDC1. Thus, our data identify the chemotherapy as a critical cause of cDC1 depletion in patients with OvC. This observation may be applied to patients affected by other cancer types that are treated by platinum-based chemotherapy. However, we also identify the IDS as a cohort that may better preserve the cDC1 subset. Indeed, patients belonging to the IDS group did not show significant loss of total lymphocytes or cDC1 when compared to HD, while chemotherapy was reducing both total lymphocytes and cDC1 frequency in the PDS group. This difference may be due to the fractionation of the therapy in the IDS group due to the surgery that is performed in between of the treatment doses. Moreover, in the IDS group the expression of PDL1 decreased upon therapy initiation and potentially rendering the cells less tolerogenic. Even though antigen uptake capacity of total CD141^+^ DC was not impaired by the pathology nor by the treatment, the capacity to respond to TLR3 ligand was partially decreased in cells taken at most of the analyzed time-points in all groups of patients and treatment did not restore this function. Moreover, for the first time, we identified a subset of cDC1 (CD86^+^ and/or ILT3^+^/ILT4^+^) potentially able to better capture exogenous antigen surrogates directly *ex vivo*. Although contradictory with a previous study showing no expression of ILT3 and ILT4 by total CD141^+^ DC (38), we used different culture and analysis conditions that may explain the observed discrepancies. Finally, the overall immune profile in blood of patients with OvC showed an increase in classical monocytes. Quantitative and phenotypic alterations in monocytes have been suggested as prognostic biomarker in patients with OvC (39, 40). Classical monocytes produce more IL-10, while non-classical monocytes constitute the subset capable of producing inflammatory cytokines in response to TLR ligands (41–43). The increased proportion of classical monocytes that we report in patients with OvC may contribute to the immunosuppression established by the tumor and, thus, to cancer progression.

Overall, our study indicates that the IDS group better preserves lymphocytes and cDC1 than the PDS group. Taking this into account when considering immunotherapy, for patients in the IDS group that usually present worse clinical conditions at diagnosis, the apheresis procedure needed to retrieve circulating DC may be better performed at the end of treatment when the patients are in a better clinical presentation. On the other hand, patients in the PDS group have a significant loss of lymphocytes and cDC1 during chemotherapy, but have usually a better clinical presentation at diagnosis; thus, for these patients, apheresis may be performed before starting standard of care chemotherapy. Of relevance, molecules such as sFlt3L and GM-CSF (44, 45) are able to increase the number of circulating DC, thus improving isolation yield (44) as well as vaccine immunogenicity (46). However, the phenotype and the function of such DC have not been characterized in patients with OvC. In addition, future vaccine strategies may include a combination of molecules aiming at improving cDC1 survival and/or response to TLR agonists and selection of subsets that have the highest potential to take up antigens. Antigen presentation and response to TLR ligands may also be improved by the metabolic reprogramming of isolated DC. Indeed, DC metabolism is altered in presence of tumor and multiple metabolic pathway have shown their importance in regulating DC development and activation (47). Recently, direct reprogramming from embryonic fibroblast has resulted in successful generation of human cDC1 (48) paving a new way for vaccination strategies. Of relevance, mechanisms of action and the clinical efficacy of multiple checkpoint inhibitors and adoptive cell therapy also depend on DC function and localization (8, 14, 15, 49–52). Thus, our observations are not only important for the efficacy of vaccine strategies but also for other immunotherapies and even other treatments that likely rely on DC functionality.

## Data availability statement

The original contributions presented in the study are included in the article/**Supplementary Material**. Further inquiries can be directed to the corresponding author.

## Ethics statement

The studies involving human participants were reviewed and approved by CHUV ethic committee, oncology department. The patients/participants provided their written informed consent to participate in this study.

## Author contributions

Conceptualization: M-GB, VS, RP, KL, SA. Methodology: M-GB, LL. Analyses: VS, LS, M-GB, WT. Funding acquisition: RP, KL, VI, MI. Supervision: VS, M-GB, RP, KL, HA. Writing – original draft: VS. Writing – review & editing: VS, M-GB, WT, RP, KL, HA, VI, MI. All authors contributed to the article and approved the submitted version.
