# Supplementary material for: Longitudinal analysis of DC subsets in patients with ovarian cancer: Implications for immunotherapy
Source: Front Immunol. 2023 Feb 10;14:1119371. doi: 10.3389/fimmu.2023.1119371 (PMC9950108; doi:10.3389/fimmu.2023.1119371)
Supplement: Supplementary file 1 [file DataSheet_1.docx]

**Supplementary Figure 1. Clinical and immunological monitoring of patients with OvC and immunological comparison with HD. (A)** Cohorts recruited and time-points of blood samples collection. **(B)** CA125 levels measured in patients with OvC before starting treatment. **C.** CA125 levels measured during treatment in patients with OvC. **(D)** Frequency of total T cells in HD and in patients with relapsing OvC undergoing treatment. **(E)** CD4 to CD8 T cell ratio in HD and in patients with OvC before and at the end of treatment. **(F)** Flow cytometry example of the proportion of monocyte populations in HD and in patients with OvC. Cumulative data of the frequency of the classical and non-classical monocyte populations in HD and in patients with OvC belonging to **(G)** IDS, **(H)** PDS, or **(I)** relapse group. **(J)** Flow cytometry example of the gating strategy used to measure the frequency of DC subsets in HD and in patients with OvC. Before gating on different DC subsets T cells, B cells and monocytes have been excluded in a dump channel. Cumulative data of the frequency of cDC1 expressing Clec9A or XCR1 in HD and in patients with OvC belonging to **(K)** IDS, (**L)** PDS, or **(M)** relapse group. **p* < 0.05. One-way ANOVA tests followed by pairwise Dunn’s tests.

**Supplementary Figure 2. Costimulatory molecules expression by total CD141^+^ DC and by cDC2 of patients with OvC and HD.**

**(A)** Flow cytometry example of the expression of CD86, PDL1 and CD80 by total CD141^+^ DC or by cDC2. (**B)** Cumulative data of the frequency of the total CD141^+^ DC expressing PDL1 at the EoT in IDS, PDS or relapse groups of patients with OvC. **p* < 0.05. One-way ANOVA tests followed by pairwise Dunn’s tests.

**Supplementary Figure 3. ILT3, ILT4, CD86 expression and dextran uptake. (A)** Representative example and **(B)** cumulative data of ILT3 and ILT4 expression by total CD141^+^ DC and cDC2. Cumulative data of the ILT3 and ILT4 expression in HD and in patients belonging to **(C)** IDS, **(D)** PDS, or **(E)** relapse group. **(F)** Flow cytometry example of the dextran uptake and CD86 by total CD141^+^ DC (dot plot) and of the dextran uptake by ILT3^+^ILT4^+^ CD141^+^ DC and ILT3^+^ILT4^-^ D141^+^ DC (histograms). **(G)** Flow cytometry example of the dextran uptake and CD86 by cDC2 (dot plot) and of the dextran uptake by ILT3^+^ILT4^-^ cDC2 and ILT3^-^ILT4^-^ cDC2 (histograms).

**Supplementary Figure 4. Poly(I:C) mediated modulation of stimulatory and inhibitory molecules in DC subsets.** Flow cytometry example of the CD86, PDL1, CD40, CD80, ILT3 and CD276 expression by **(A)** total CD141^+^ DC or **(B)** cDC2 without (*i.e.* Unstim) and with stimulation by Poly(I:C).
